# The Spatial Signature of Glioblastoma: A Statistical Re-Assessment of Anatomical Distribution Based on Methylation Subtypes

**DOI:** 10.3390/cells15020175

**Published:** 2026-01-19

**Authors:** Tim Herrmann, Claire Delbridge, Michael Griessmair, Julian Canisius, Meike Mitsdoerffer, Denise Bernhardt, Isabel C. Hostettler, Chiara Negwer, Igor Yakushev, Bernhard Meyer, Friederike Schmidt-Graf, Stephanie E. Combs, Jan S. Kirschke, Benedikt Wiestler, Marie-Christin Metz

**Affiliations:** 1 Department of Neuroradiology, School of Medicine and Health, Technical University of Munich, 81675 Munich, Germanyb.wiestler@tum.de (B.W.); marie.metz@tum.de (M.-C.M.); 2 Institute of Pathology, School of Medicine and Health, Technical University of Munich, 81675 Munich, Germany; 3 Department of Diagnostic, Interventional and Pediatric Radiology, Inselspital Bern, University of Bern, 3010 Bern, Switzerland; 4 Department of Neurology, School of Medicine and Health, Technical University of Munich, 81675 Munich, Germany; 5 Department of Radiation Oncology, School of Medicine and Health, Technical University of Munich, 81675 Munich, Germany; 6 Department of Neurosurgery, School of Medicine and Health, Technical University of Munich, 81675 Munich, Germany; 7 Department of Neurosurgery, HOCH Health Ostschweiz, Cantonal Hospital St. Gallen, 9007 St. Gallen, Switzerland; 8 Department of Nuclear Medicine, School of Medicine and Health, Technical University of Munich, 81675 Munich, Germany; 9 TranslaTUM, Technical University of Munich, 81675 Munich, Germany

**Keywords:** glioblastoma, methylation analysis, MGMT methylation status

## Abstract

Precise molecular characterization of glioblastoma (GB) is fundamental for accurate risk stratification and therapeutic planning. DNA methylation profiling reliably identifies key molecular features, including O(6)-methylguanine-DNA methyltransferase (MGMT) promoter methylation status and specific molecular subtypes, such as receptor tyrosine kinase (RTK) I and II, and the mesenchymal (MES) subtype. In this study, we investigated the hypothesized correlation between these molecular profiles and preferential tumor locations, which could reveal a link to underlying tumor biology. We analyzed 227 GB patients characterized by DNA methylation profiling. To map significant clusters of tumor occurrence across subtypes and subcomponents, we performed voxel-wise analysis of differential involvement, utilizing 500 permutations to correct for multiple comparisons. While uncorrected frequency differential maps suggested localization tendencies for the RTK I, RTK II, and MES subtypes, stringent statistical correction revealed only one robust association: the non-enhancing component of MES tumors showed significant clustering in the left frontal lobe, the insula, and the temporal lobe. Contrary to prior literature, we observed no significant hemispheric preference regarding MGMT promoter methylation status. Our findings challenge prior assumptions regarding the spatial distinctiveness of GB subtypes and highlight the need to further elucidate the mechanisms governing tumorigenesis and spatial growth patterns.

## 1. Introduction

Glioblastoma (GB), the most common form of malignant primary brain tumor, remains a major therapeutic challenge because it infiltrates surrounding brain tissue extensively and exhibits high molecular and clinical variability. Although research and treatment approaches have advanced, patient outcomes remain poor. Only about 7% of individuals survive beyond 5 years, and disease progression differs significantly between patients [1]. Due to the considerable molecular heterogeneity of brain tumors, the World Health Organization (WHO) classification of central nervous system (CNS) tumors, revised in 2021, now includes molecular patterns alongside histological features, given their powerful prognostic value [2].

GB outcomes are significantly influenced by tumor localization, as demonstrated in previous studies. Central regions, such as the corpus callosum and basal ganglia, are associated with markedly worse survival, whereas tumors in less central brain areas may predict longer survival trajectories [3].

In recent years, advances in the molecular characterization of GB have raised the question of whether different molecular subtypes show distinct spatial distributions in the brain. Identifying these clusters may aid in the initial risk stratification for patients, inform treatment decisions, and enhance prognostic models [4,5].

One of the key molecular features of GB is the methylation status of the DNA repair enzyme O(6)-methylguanine-DNA methyltransferase (MGMT). This enzyme antagonizes the genotoxic effect of alkylating agents. Patients with MGMT silencing, who are treated with alkylating agent chemotherapy, such as temozolomide, have a favorable outcome [6].

Ellingson et al. found that MGMT-methylated tumors occurred significantly more often in the left hemisphere, with a pronounced cluster in the left temporal lobe. In contrast, MGMT-unmethylated tumors were preferentially located in the right hemisphere, particularly involving the right temporal lobe, basal ganglia, and subventricular zone. Patients whose contrast-enhancing tumors (CET) involved the MGMT-methylated cluster in the left temporal lobe had markedly longer overall survival compared to patients with tumors elsewhere [7]. However, later studies found conflicting results [8,9,10].

Further advances in genome-wide DNA methylation profiling have substantially improved the molecular classification of GB. This approach enables a more refined stratification into distinct methylation-defined subclasses, of which the Receptor Tyrosine Kinase (RTK) I, II, and mesenchymal (MES) subtypes are the most prevalent groups in adults. These subclasses are characterized by reproducible epigenetic signatures and associated copy-number profiles, providing a level of biological and diagnostic resolution that clearly exceeds that which can be achieved by conventional histopathology alone [11].

Tejada Neyra et al. systematically assessed whether classical DNA methylation subclasses of GB—RTK I, II, and MES—show specific anatomical predilections, using voxel-based lesion–symptom mapping (VLSM). Their analysis found no statistically significant spatial localization for any of the isocitrate dehydrogenase (IDH)-wildtype methylation subclasses after stringent multiple testing correction [12]. In contrast, Foltyn-Dumitru et al. provided evidence that these subclasses do exhibit specific anatomical predilections, challenging the previously held assumption of their spatial randomness. They used a support vector regression-based lesion–symptom mapping approach to identify associated anatomical brain regions based on methylation subtypes. They observed that MES subtypes exhibited a strong left hemispheric predilection, particularly involving the insula and posterior temporal lobe. RTK I tumors were predominantly mapped to the right frontal regions, whereas RTK II tumors showed three dominant clusters, particularly within the left hemisphere [13].

The therapeutic impact of these anatomical and molecular features is substantiated by Wick et al., who demonstrated that RTK II subclasses, especially when also showing MGMT promoter methylation, are statistically more likely to be located in central/periventricular regions of the brain. This subgroup of patients does not benefit from standard radiotherapy but benefits distinctly from temozolomide-based chemotherapy. This finding highlights the importance of integrating molecular and anatomical features for personalized GB management [14].

The immune microenvironment of distinct methylation subclasses of GB was further characterized by Dejaegher et al., who illustrated that MES and RTK subtypes, especially RTK II, tend to arise in highly vascularized and periventricular regions. These areas were also associated with higher infiltration of immune cells (T cells), which may impact prognosis and inform immunotherapeutic strategies [15].

Surgical management may also benefit from this molecular–anatomical understanding. It has been demonstrated that RTK II and MES subgroups show a greater survival benefit from gross total resection in certain methylation-defined patient groups [16].

Additionally, RTK II methylation subclass is associated with tumors that show proximity to regions responsible for seizure generation, offering insight into symptom development and potential preoperative management [17].

Taken together, these studies demonstrate a general consensus regarding the potential prognostic and clinical importance of a combined molecular and anatomical approach in patients with GB. However, there is conflicting evidence regarding the anatomical distribution in relation to its molecular characteristics.

This study aims to clarify whether different GB subclasses and MGMT promoter methylation status are associated with preferential brain localizations, utilizing a state-of-the-art image post-processing pipeline, as well as an established voxel-wise analysis that focuses on different tumor subregions.

## 2. Materials and Methods

### 2.1. Patient Data

This was a retrospective analysis of an ongoing patient cohort of brain tumor patients, approved by our local institutional review board (IRB #340/16S). From this, we selected patients who had been diagnosed with GB, IDH-wildtype, by state-of-the-art neuropathological and molecular methods between January 2020 and March 2024, as per the WHO 2021 classification for tumors of the CNS [2].

To be included in our analysis, the availability of our standard magnetic resonance imaging (MRI) protocol in the preoperative setting was required, which included at least 3D-FLAIR (fluid-attenuated inversion recovery) and/or axial T2 TSE (turbo spin echo), as well as a T1 sequence before and after the administration of a gadolinium-based contrast agent. Furthermore, access to an advanced 850k methylation analysis of the intraoperatively acquired tumor tissue was necessary to obtain detailed molecular information about the tumor.

### 2.2. Image Acquisition and Postprocessing

Since we only examined in-house imaging data, all MRI was performed using a Philips 3 Tesla whole-body scanner (Achieva or Ingenia, Philips, Best, The Netherlands) or a Siemens Verio 3 Tesla whole-body scanner (Siemens, Erlangen, Germany). The typical protocol consisted of an isotropic FLAIR sequence (voxel size—1 mm^3^, echo time (TE)—269 ms, repetition time (TR)—4800 ms, inversion time (TI)—1650 ms), isotropic T1-weigthed turbo field echo (TFE) (voxel size—1 mm^3^, TE—4 ms, TR—9 ms) before and after contrast, and axial T2-weighted sequence (voxel size—0.36 × 0.36 × 4 mm^3^, TE—87 ms, TR—3396 ms). The additional dynamic susceptibility contrast perfusion imaging and diffusion tensor imaging that we usually acquire were not evaluated in this study.

All images were rigidly co-registered into the SRI24 atlas space (Stanford Research Institute, SRI) using NiftyReg and skullstripped using HD-BET [18,19].

Afterwards, we automatically segmented tumor subregions into CET, necrosis, and edema by applying the BraTS.Toolkit [20]. This freely available tool ensembles various state-of-the-art image segmentation algorithms. In cases where T2-weighted or T2-FLAIR images are missing, these sequences were artificially synthesized using a GAN-based approach to enhance automated segmentation. Segmentation performance with artificial T2/FLAIR images was validated on a multi-institutional glioma cohort in our original publication [21]. All segmentations were individually checked and manually corrected if needed using ITK-Snap (version 3.6.0) [22]. Illustrated examples of the registration process, from native patient anatomy to SRI atlas space, plus an illustration of the tumor segmentation, can be found in the Supplementary Material (Figures S1–S3).

### 2.3. Molecular Profiling

All tissue samples from the initial tumor resection were formalin-fixed and paraffin-embedded. Afterwards, they were comprehensively examined using the classical neuropathological standard. In addition to conventional histology and immunohistochemistry, an 850k methylation analysis was performed on extracted DNA using Illumina EPIC 850k Methylation Array BeadChip (Illumina, Inc., San Diego, CA, USA) and evaluated using the Brain Tumor classifier of the DKFZ and the University of Heidelberg [11,23]. Molecular markers of interest included MGMT methylation status and predicted tumor subtype.

### 2.4. Spatial Localization Analysis

For spatial localization analysis, patients were stratified by gender, molecular subtype, and MGMT promoter methylation status (methylated vs. unmethylated). To minimize potential confounders, we calculated the mean volumes of whole tumor, tumor core, and non-enhancing/edema for each molecular subgroup and MGMT methylation status and performed Kruskal–Wallis and Mann–Whitney-U tests to check for significant differences.

Next, we examined the hemispheric distribution to characterize left–right anatomical patterns across molecular subtypes. Therefore, we computed the spatial center of mass for each patient’s tumor as part of a whole-tumor analysis and a tumor core analysis (which only included necrotic and enhancing tumor parts). Hemispheric classification was based on the location of the center of mass in relation to the anatomical midline. A chi-square test was conducted to assess significant differences between subgroups.

Then, we performed a simple voxel-wise frequency calculation. For each voxel in SRI space, we calculated the percentage of patients with tumor presence:

F(v) = (N_tumor(v)/N_total) × 100,

where N_tumor(v) = number of patients with tumor at voxel v and N_total = total patients’ count in molecular subtype group.

For each molecular subtype, the maximum frequency per analysis (the highest percentage across all voxels) was calculated, as well as voxel counts above clinically relevant thresholds (10% and 20%).

Based on these frequency maps, we calculated frequency differential maps between two molecular subtypes, where each voxel indicates the difference in the percentage of patients with tumors at this voxel, providing a first visual impression of the tumor distributions.

To test for statistical differences in the spatial localization of molecular subtypes, we performed an analysis of differential involvement (ADIFFI), similar to that described by Ellingson et al. [7].

Here, the null hypothesis is that there is no spatial difference in tumor distribution between molecular subtypes, while the alternative hypothesis proposes significant clustering differences. A Voxel-wise Fisher’s exact test was conducted for group comparisons. To correct for multiple comparisons, 500 permutations were performed for robust statistical inference for each sub-analysis. This included random reassignment of molecular labels while preserving group sizes. This generated a null distribution for cluster-level statistics. The raw, uncorrected *p*-value threshold was defined by *p* < 0.005. The minimum cluster size was determined empirically from the null distribution. The analysis’s output included the number of significant clusters after correction, the minimum raw *p*-value across all voxels, and the total number of significantly different voxels.

**Figure 1:**
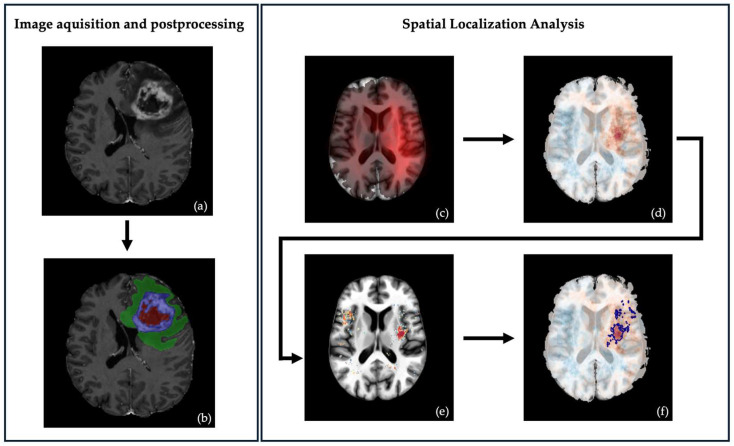
Illustrating the workflow from image acquisition, postprocessing, and spatial localization analysis: (**a**) MRI, T1 contrast-enhanced sequence: typical GB phenotype with central necrosis, contrast enhancement, and perifocal edema. (**b**) Same tumor after automated segmentation: (red) central necrosis, (blue) contrast enhancement, (green) perifocal edema. (**c**) Frequency of distinct methylation subtype per voxel. (**d**) Differential frequency map, prevalence per voxel between two subtypes. (**e**) Voxel-wise raw *p*-value of the *t*-test between two subtypes. (**f**) Significant clusters are shown in dark blue on the differential map after permutation analysis.

Our workflow is illustrated in Figure 1.

All analyses were performed using Python 3.10 or later, with scientific computing libraries. Core packages included nibabel (version 5.3.2), scipy (version 1.15.3), and matplotlib (version 3.10.3). A high-memory computing environment was necessary for large-scale permutation testing, as well as parallel processing capabilities for efficiency.

A generative artificial intelligence (Claude Sonnet 4 via GitHub Copilot, version 0.33.1) was utilized to aid in code production and enhancement, improving efficiency. The reliability of AI-generated code sections was evaluated by incorporating test cases.

## 3. Results

### 3.1. Descriptive Features of the Study Cohort 

A total of 227 patients met the inclusion criteria based on clinical, epigenetic, and MRI data, with 64.8% male and 35.2% female. DNA methylation profiling revealed MES subtype as the predominant molecular tumor subtype (37.4%), followed by RTK I (30.4%), RTK II (23.8%), and others (8.4%; MYCN, midline, H3 G34 mutant, H3 K27-altered).

MGMT promoter methylation status was equally distributed, with 48.9% of patients showing methylated and 50.2% showing unmethylated promoter status. Due to missing neuropathology reports, MGMT promoter methylation status was unavailable for two patients (0.9%), who were excluded from this part of the analysis. Detailed demographic information can be found in Table 1.

### 3.2. Tumor Volumes and Hemispheric Distribution

Tumor volumes did not differ significantly between molecular subgroups, with the MES subtype showing the largest mean tumor volume (104 cm^3^), followed by the RTK I subtype (100 cm^3^) and the RTK II subtype (88 cm^3^). Also, MGMT promoter methylated and unmethylated tumors exhibited similar mean tumor volumes (96 vs. 103 cm^3^, Table 2).

There was a right-hemispheric dominance in our patient cohort, with 56.4% of tumors having their center of mass in the right hemisphere versus 43.6% in the left hemisphere across all molecular subgroups. However, this difference was not significant (*p* = 0.054).

The chi-square test revealed no significant difference in the hemispheric distribution of tumors between female and male patients (*p* = 1.00).

Stratified by molecular subtype, the MES subtype showed a slight tendency towards the right hemisphere (66%), while RTK I and RTK II subtypes were equally distributed between both hemispheres (Table 2). There was no statistically significant difference between the subgroups (*p* = 0.21).

Additionally, tumors with unmethylated MGMT promoter status showed a slight but non-significant predominance in the right hemisphere (58.5%), whereas tumors with methylated MGMT promoter methylation status were more evenly distributed between the right (54.1%) and left hemispheres (45.9%).

### 3.3. Voxel-Wise Analysis of Differential Involvement

**Figure 2:**
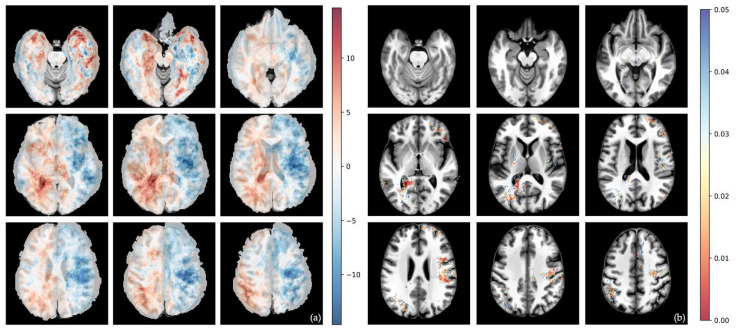
Voxel-wise distribution of MGMT-methylated vs. -unmethylated GB. (**a**) Differential frequency per voxel for whole-tumor volume in percent. Red—higher frequency in methylated tumor; blue—higher frequency in unmethylated tumor. (**b**) Voxel-wise raw *p*-value of *t*-test for whole-tumor volume. The color bar shows *p*-values from 0.00 (red) to 0.05 (blue).

**Figure 3:**
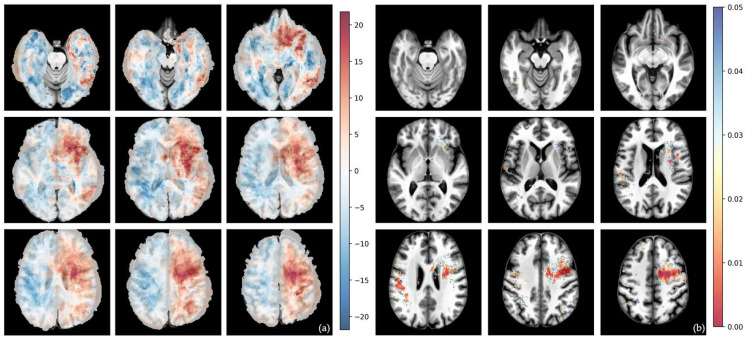
Voxel-wise distribution of MES vs. RTK I subtype. (**a**) Differential frequency per voxel for whole-tumor volume in percent. Red—higher frequency in MES subtype; blue—higher frequency in RTK I subtype. (**b**) Voxel-wise raw *p*-value of *t*-test for whole-tumor volume. The color bar shows *p*-values from 0.00 (red) to 0.05 (blue).

**Figure 4:**
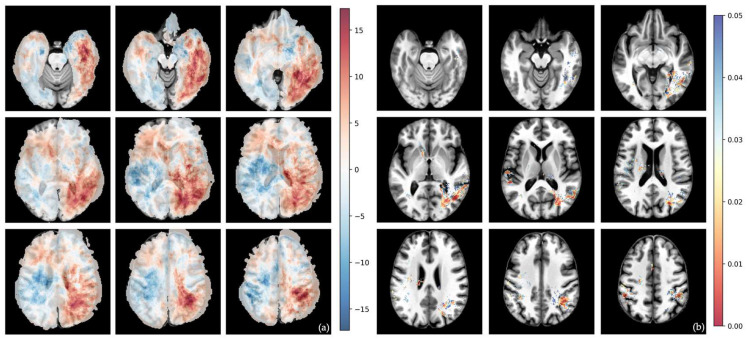
Voxel-wise distribution of MES vs. RTK II subtype. (**a**) Differential frequency per voxel for whole-tumor volume in percent. Red—higher frequency in MES subtype; blue—higher frequency in RTK II subtype. (**b**) Voxel-wise raw *p*-value of *t*-test for whole-tumor volume. The color bar shows *p*-values from 0.00 (red) to 0.05 (blue).

**Figure 5:**
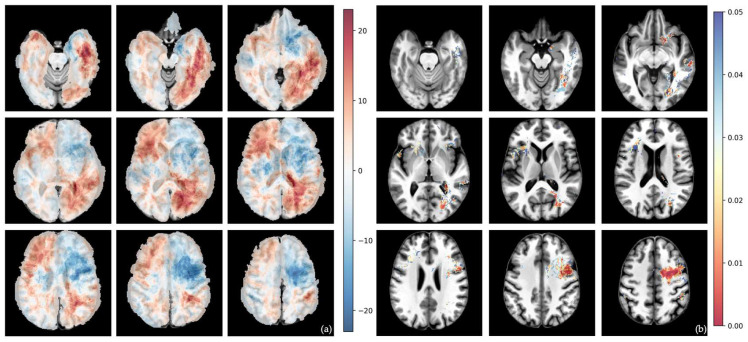
Voxel-wise distribution of RTK I vs. RTK II. (**a**) Differential frequency per voxel for whole-tumor volume in percent. Red—higher frequency in RTK I subtype; blue—higher frequency in RKTII subtype. (**b**) Voxel-wise raw *p*-value of *t*-test for whole-tumor volume. The color bar shows *p*-values from 0.00 (red) to 0.05 (blue).

The differential voxel-wise frequency maps and their corresponding voxel-wise raw *p*-values from Fisher’s exact test for whole tumor volumes are shown in Figure 2, Figure 3, Figure 4 and Figure 5.

Contrary to the center of mass analysis, the differential maps suggested a higher prevalence of tumors with unmethylated MGMT promoter status in the left hemisphere, although there were only a few significant clusters even before permutation analysis, and none afterwards.

Compared to RTK II and MES tumor types, RTK I tumors show the highest prevalence in the frontal lobes (especially in the right frontal lobe) (Figure 3 and Figure 5). However, no significant cluster was identified.

The MES subtype showed a clear tendency towards the left frontal and temporal lobes (Figure 3 and Figure 4).

**Figure 6:**
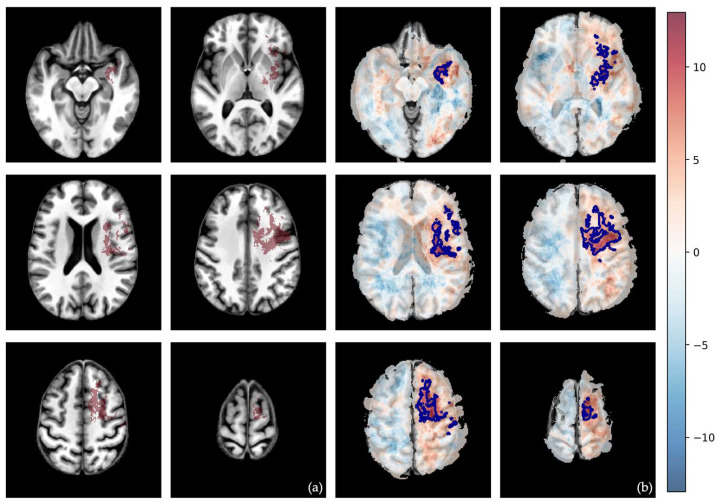
Illustration of the only significant cluster following permutation analysis. The non-enhancing tumor of the MES subtype is significantly more frequently located in the left frontal and temporal lobes, including the insula, compared to the RTK I subtype. The cluster is shown in red on anatomical slices of an atlas brain on the left (**a**), and in blue overlaid on the different slices of the frequency differential map between MES and RTK I subtype (**b**). Red—higher frequency in MES subtype; blue—higher frequency in RTK I subtype.

After permutation-correction for multiple comparisons (with 500 permutations), only one significant cluster survived. This cluster of 35,971 voxels, located in the left hemisphere primarily in the insular and periventricular regions, shows a 239% higher frequency of non-enhancing tumor/edema in the MES subtype compared to the RTK I subtype (Figure 6). In other words, in this specific cluster, the likelihood of tumor occurrence of non-enhancing tumor/edema is 239% higher for the MES subtype than for the RTK I subtype. All other comparisons showed no statistically significant clusters after correction for multiple comparisons. Detailed information about the voxel-wise analysis can be found in Table S1 in the Supplementary Material. All differential maps and the raw p-value maps for the different tumor subcompartments (tumor-core, non-enhancing tumor/edema, and whole tumor) are also listed in the Supplementary Material, Figures S4–S7.

## 4. Discussion

Understanding the spatial preferences of molecular subgroups of GB would be highly interesting for initial risk assessment and therapy planning in GB patients. Additionally, it may provide insight into a more detailed understanding of GB ontogeny and growth. To date, prior work has yielded conflicting evidence on this topic.

Here, we analyzed a 227-patient cohort of IDH-wildtype GB (as per the 2021 WHO classification) with standardized imaging and segmentation, as well as molecular classification via 850k EPIC methylation analysis.

We were interested in the potential spatial clustering of MGMT promoter-methylated versus non-methylated GB and of the main molecular subtypes, including MES, RTK I, and RTK II, in the brain.

After rigorous voxel-wise analysis with empirical cluster-size thresholds and 500 permutations to account for multiple comparisons, only one spatial effect survived: a large left frontal and temporal cluster with an insular/periventricular focus, where MES tumors show a markedly higher frequency of non-enhancing tumors than RTK I. Additionally, we can only report non-significant growth tendencies, which are partly in line with prior assumptions.

### 4.1. Exclusion of Tumor Volume or Lateralization Bias

Notably, in our cohort, tumor volumes of the different sub-compartments (whole tumor, tumor core, and non-enhancing tumor/edema) did not differ significantly between methylation subtypes or MGMT groups, which aligns with prior studies [13]. Also, gross hemispheric distributions, characterized by the localization of the center-of-mass in relation to the interhemispheric fissure, were broadly similar. Therefore, a significant cluster in our analysis reflects a specific regional preference rather than global volume or laterality bias.

However, it is noteworthy that our overall cohort showed a borderline dominance in the right hemisphere (56% vs. 44%, *p* = 0.054). Recent studies have found no significant difference in the prevalence of GB between the right and left hemispheres [4,24,25]. However, even in the latest meta-analysis from 2024, no study was included that investigated only IDH wild-type GB, according to the 2021 WHO classification [25]. Therefore, it would be interesting to explore this observation in more detail through larger studies.

### 4.2. MGMT-Methylated and -Unmethylated GB Do Not Differ in Spatial Localization

There has been conflicting evidence regarding the lateralization and spatial clustering of MGMT-methylated versus -unmethylated GB, which can be largely attributed to the heterogeneous study populations and varying statistical methods employed.

In two large studies, Ellingson et al. reported a clear lateralization towards the left hemisphere for MGMT-methylated GB, whereas MGMT-unmethylated tumors showed lateralization to the right hemisphere [7,26]. Other studies have found no significant difference in tumor lateralization between the two groups [8,9,10].

In line with the latter, we did not find a significant association between MGMT status and hemisphere via center-of-mass analysis (*p* = 0.56), although there was a slight tendency towards the right hemisphere for the unmethylated subgroup (59% vs. 41%). However, in contrast to this, visual inspection of the differential frequency maps revealed a reverse tendency, with a slightly higher prevalence of MGMT-methylated GB in the right hemisphere. Nevertheless, there were no significant clusters for whole-tumor volume, tumor core, and non-enhancing tumor/edema, respectively. This led us to the conclusion that MGMT-methylated and -unmethylated tumors do not differ in spatial localization, supporting our prior work, where we did not find an association between GB contact with the subventricular zone and MGMT methylation status [27]. Since, to the best of our knowledge, Incekara et al. are the only ones who have only included IDH wild-type GB in their analysis, while all other previously mentioned studies also included IDH-mutated GB, it is reasonable that our results align most closely with theirs [10].

### 4.3. MES GB Shows a Clear Tendency Towards the Left Frontal and Temporal Lobes

Advanced DNA methylation profiling enables the classification of GB into multiple subclasses, which differ in their clinical behavior and response to treatment. RTK I, RTK II, and MES types are the most common, and they do not differ in their overall survival outcomes; however, they exhibit different responses to treatment strategies, such as gross total resection or re-resection [16]. Therefore, it will likely be an important field of research in the future to identify these molecular subclasses, investigate their distinct mechanisms of tumorigenesis and tumor growth, and ultimately design molecularly informed, personalized treatment regimens.

Only a few studies have investigated the association between molecular subtype and spatial localization in the brain to date, using various voxel-wise analysis methods [12,13,26].

Similarly to studies on the localization preference of different MGMT statuses, these studies have produced conflicting results.

Here, we utilized a rigorous voxel-wise ADIFFI pipeline, consisting of Fisher’s exact test, 500 permutations per analysis, and empirical cluster-size thresholds, to analyze different tumor subareas, namely, the whole tumor, tumor core, and non-enhancing tumor/edema. Following this strict statistical analysis, only one statistically significant cluster was observed:

Compared to the RTK I subtype, the MES subtype exhibited a significant cluster in the left frontal and temporal lobes, including the insular region, as well as large periventricular areas. However, while we saw a clear tendency for the whole-tumor volume (in the differential frequency maps), the cluster was only significant for the non-enhancing tumor/edema part. Foltyn-Dumitru et al. made very similar observations, with the only difference being that they investigated the entire tumor volume and found a similar, significant cluster in the left frontal/temporal lobes, and particularly in the left insula, for the MES subtype as well [13]. This discrepancy may stem from a completely different technical approach, as they employed multivariate support vector regression lesion-symptom mapping. Our observation can be seen as an extension of this finding, indicating that, within this common territory, MES also has disproportionately more edema than RTK I. The fact that our conservative, simpler, yet more transparent approach yields a similar result suggests that the observed cluster is not driven by overfitting or model flexibility, but rather by a strong signal. While these results do not allow for clinical inference, left insular and periventricular tumor growth may be indicative of the MES subtype, a hypothesis that warrants further investigation incorporating additional imaging markers.

In contrast, our analysis does not confirm other observations made by Foltyn-Dumitru et al. [13]. While visual inspection of our frequency differential maps suggests that the RTK I subtype shows the highest prevalence in the frontal lobes (especially in the right frontal lobe), no cluster survived permutation analysis. Additionally, the RTK II subtype only showed a visual preference for the left frontal lobe in the differential frequency map compared to the RTK I subtype. However, no statistically significant cluster was observed here either.

Therefore, this part of the analysis aligns with Tejada Neyra et al., who also did not observe any tumor localization predilection for the different methylation classes using univariate voxel-based lesion mapping [12].

### 4.4. Limitations

Some limitations of our study have to be considered when interpreting the results:

First, this is a retrospective single-center study. While this provides a homogeneous patient population and ensures consistent image quality, external validation in a larger dataset is needed. Second, as stated before, contrary to other studies, we used a univariate, cluster-based approach rather than multivariate machine learning models, which can capture more complex spatial patterns. However, it is noteworthy that our significant finding is robust even under conservative univariate correction. Lastly, potential unmeasured confounders, such as detailed vascular or immunologic differences, could have influenced tumor growth behavior, especially in the case of edema.

## 5. Conclusions

In conclusion, we can confirm a local preference for the MES subtype in the left frontal and temporal lobes as well as in the insula. Whether this local preference is attributed to increased immune activity in the insula remains to be determined [15]. Since we only observed significance for the non-enhancing tumor/edema volume, it will be interesting to explore which biological factors of MES GB drive a more extensive peritumoral edema in insular and periventricular tissue, such as a more invasive phenotype with early blood–brain barrier disruption, or microenvironmental and immune differences.

Other than that, with our strict univariate and conservative statistical approach, we cannot confirm any significant localization differences for the main methylation subtypes of GB on a voxel-wise level. We postulate that MGMT-methylated and -unmethylated tumors are evenly distributed throughout the brain and do not exhibit significant clustering. Our findings encourage further research on the exact mechanisms of tumorigenesis and tumor growth, potentially integrating a more comprehensive approach that includes microenvironmental and immune factors.

## Figures and Tables

**Table 1:** Characteristics of the study population.

Patients Characteristics	Total (*n* = 227)
Age (years old) at time of initial diagnosis (mean)	
male	64.2
female	66.9
all	65.2
Gender (absolute and in percent)	
male	147 (64.8)
female	80 (35.2)
all	227
MGMT Methylation status (absolute and in percent)	
methylated	111 (48.9)
unmethylated	114 (50.2)
missing	2 (0.9)
Molecular subtype (absolute and in percent)	
MES	85 (37.4)
RTK I	69 (30.4)
RTK II	54 (23.8)
Other	19 (8.4)

**Table 2:** Volumetrics and hemispheric distribution.

Parameter	MES	RTK I	RTK II	*p*-Value	MGMT-Methylated	MGMT-Unmethylated	*p*-Value
Volume (mean in cm^3^)							
Whole tumor	104	100	88	0.21	96	103	0.51
Tumor core	35	38	30	0.12	33	36	0.53
Non-enhancing tumor	68	62	58	0.44	63	67	0.76
Hemispheric Distribution							
(absolute and in percent)							
Right-hemispheric	56 (65.9)	35 (50.7)	26 (48.1)	0.20	60 (54.1)	67 (58.8)	0.56
Left-hemispheric	29 (34.1)	34 (49.3)	28 (51.9)		51 (45.9)	47 (41.1)	

## Data Availability

The clinical and imaging data are not publicly available due to privacy restrictions.

## Supplementary Materials

The following supporting information can be downloaded at: https://www.mdpi.com/article/10.3390/cells15020175/s1, Table S1: ADIFFI_MultiAnalysis_Results, Figures S1–S3: Examples of image registration to SRI space and segmentation, Figures S4–S7: Differential map and raw *p*-value for all subcompartments.

## Abbreviations

The following abbreviations are used in this manuscript:

ADIFFI	Analysis of differential involvement
CET	Contrast-enhancing tumor
CNS	Central nervous system
FLAIR	Fluid attenuated inversion recovery
GB	Glioblastoma
IDH	Isocitrat-dehydrogenase
MES	Mesenchymal
MGMT	O(6)-methylguanine-DNA methyltransferase
RTK	Receptor tyrosine kinase
SRI	Stanford Research Institute
TE	Echo time
TFE	Turbo field echo
TI	Inversion time
TR	Repetition time
TSE	Turbo spin echo
VLSM	Voxel-based lesion-symptom mapping
WHO	World Health Organization
