# Supplementary figures and images for: The Spatial Signature of Glioblastoma: A Statistical Re-Assessment of Anatomical Distribution Based on Methylation Subtypes
Source: Cells. 2026 Jan 19;15(2):175. doi: 10.3390/cells15020175 (PMC12840343; doi:10.3390/cells15020175)

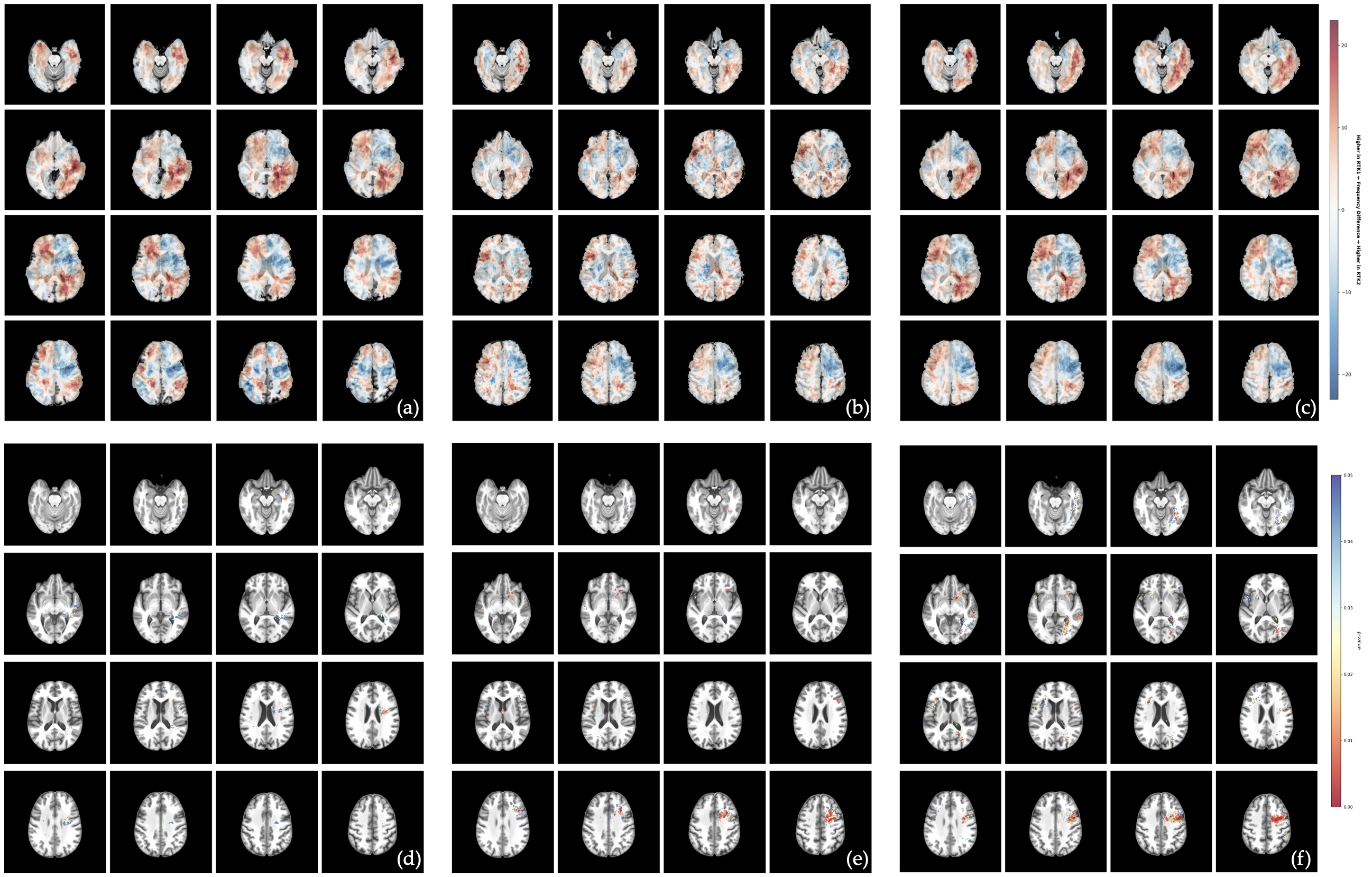

Supplement: Supplementary file 1 [file cells-15-00175-s001.zip › Figure S4_RTKI vs. RKTII_Differential map & Raw p-value_(all subcompartments).png]

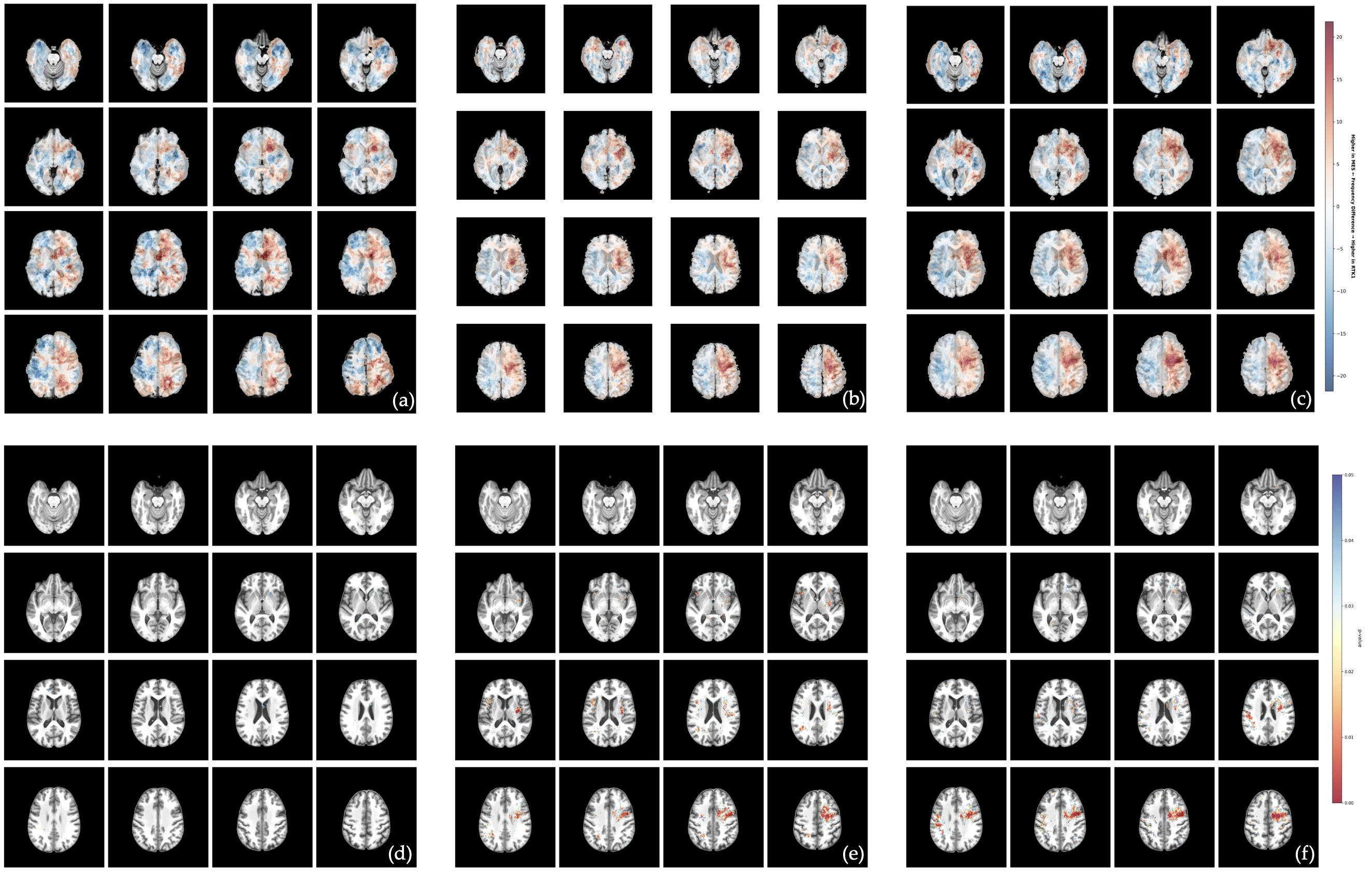

Supplement: Supplementary file 1 [file cells-15-00175-s001.zip › Figure S5_MES vs. RTKI_Differential map & Raw p-value_(all subcompartments).png]

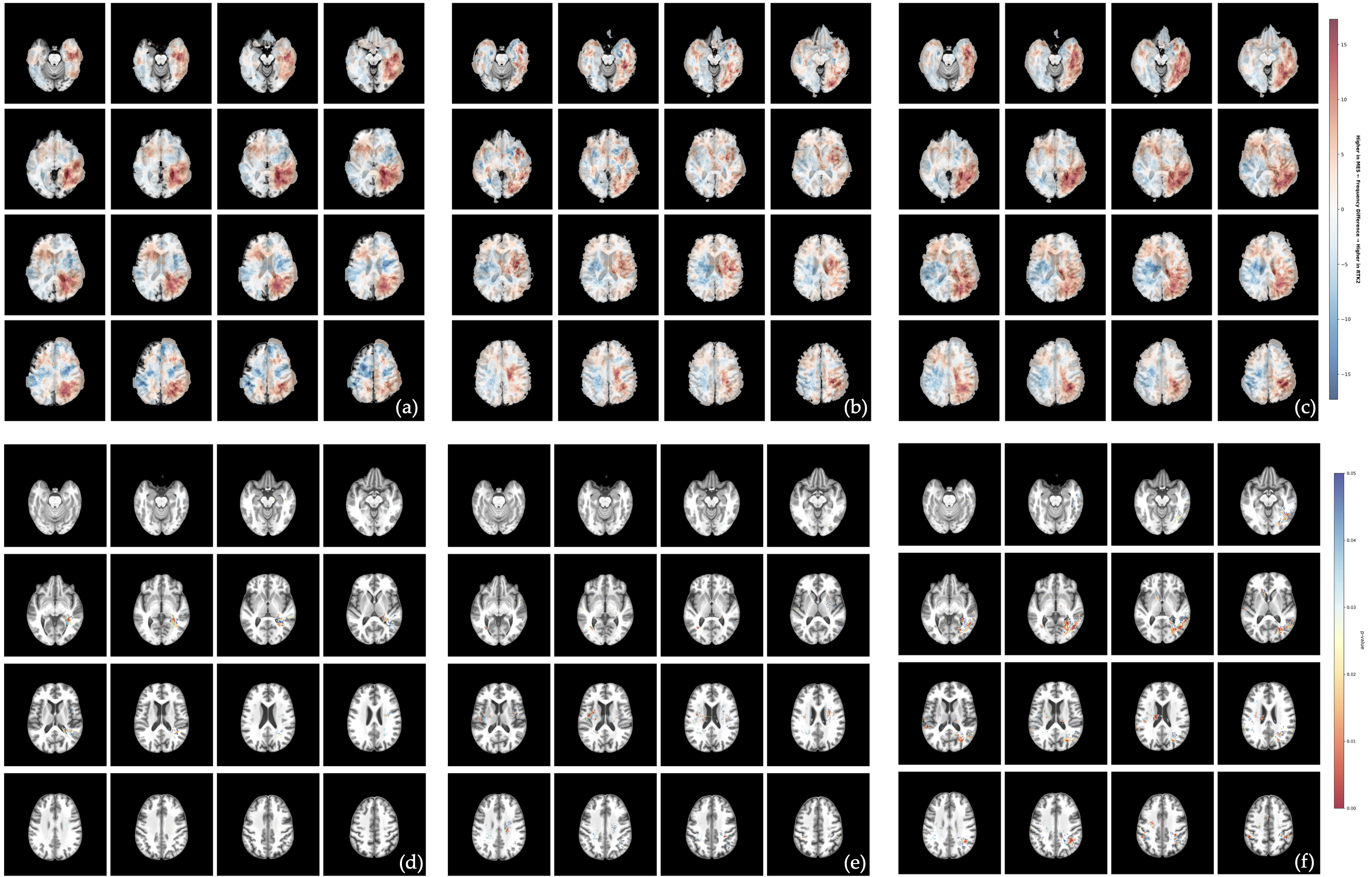

Supplement: Supplementary file 1 [file cells-15-00175-s001.zip › Figure S6_MES vs. RTKII_Differential map & Raw p-value_(all subcompartments).png]

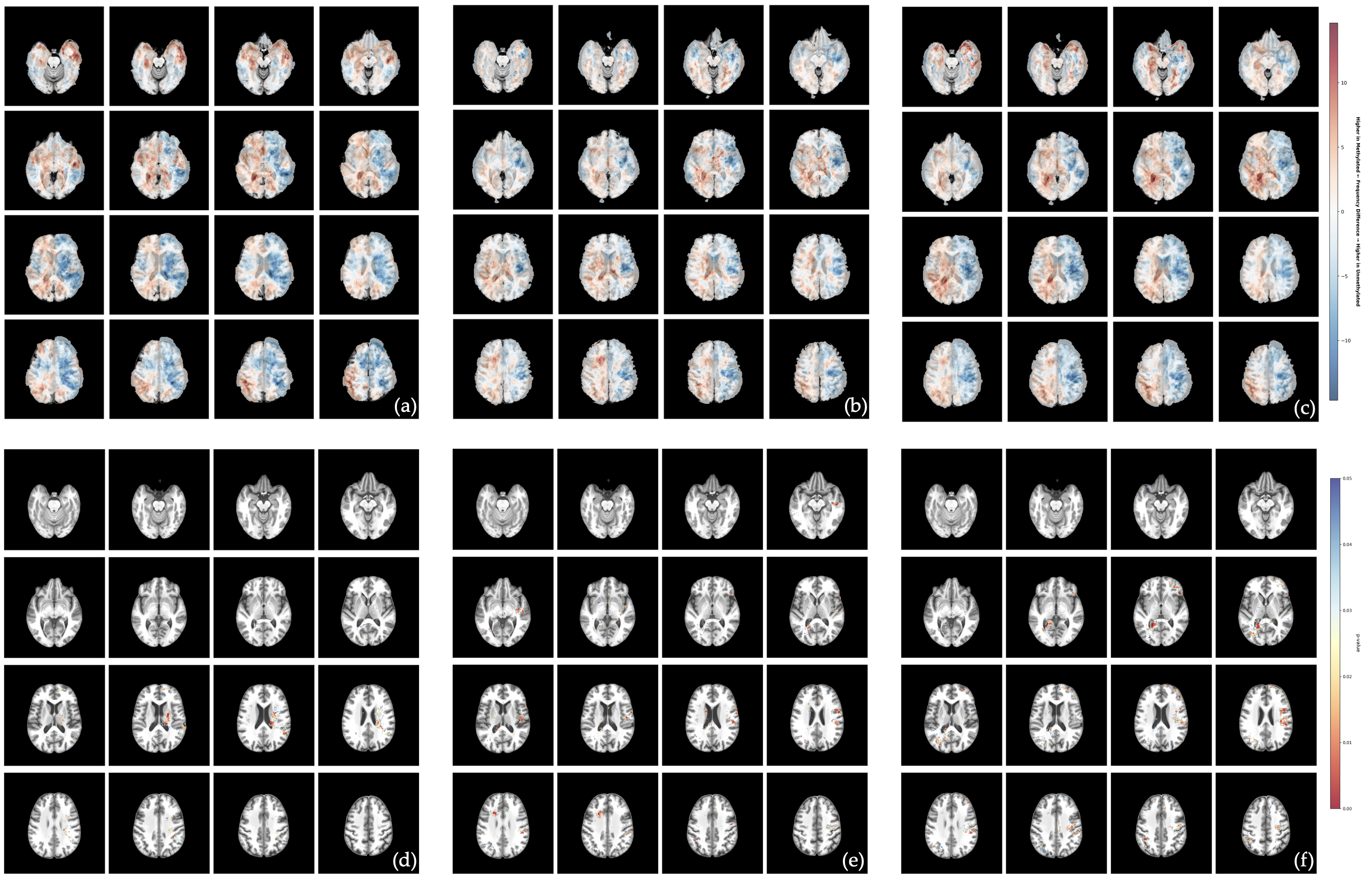

Supplement: Supplementary file 1 [file cells-15-00175-s001.zip › Figure S7_MGMT methylated vs. unmethylated_Differential map & Raw p-value_(all subcompartments).png]
